# Energy Expenditure in People with Diabetes Mellitus: A Review

**DOI:** 10.3389/fnut.2016.00056

**Published:** 2016-12-22

**Authors:** Nathan Caron, Nicolas Peyrot, Teddy Caderby, Chantal Verkindt, Georges Dalleau

**Affiliations:** ^1^Laboratoire IRISSE, UFR des Sciences de l’Homme et de l’Environnement, Université de la Réunion, Le Tampon, La Réunion, France

**Keywords:** type 2 diabetes, metabolic cost, physical activity, guideline, sensor

## Abstract

Physical activity (PA) is an important non-therapeutic tool in primary prevention and treatment of diabetes mellitus (DM). To improve activity-based health management, patients need to quantify activity-related energy expenditure and the other components of total daily energy expenditure. This review explores differences between the components of total energy expenditure in patients with DM and healthy people and presents various tools for assessing the energy expenditure in subjects with DM. From this review, it appears that patients with uncontrolled DM have a higher basal energy expenditure (BEE) than healthy people which must be considered in the establishment of new BEE estimate equations. Moreover, studies showed a lower activity energy expenditure in patients with DM than in healthy ones. This difference may be partially explained by patient with DMs poor compliance with exercise recommendations and their greater participation in lower intensity activities. These specificities of PA need to be taken into account in the development of adapted tools to assess activity energy expenditure and daily energy expenditure in people with DM. Few estimation tools are tested in subjects with DM and this results in a lack of accuracy especially for their particular patterns of activity. Thus, future studies should examine sensors coupling different technologies or method that is specifically designed to accurately assess energy expenditure in patients with diabetes in daily life.

## Introduction

In recent decades, a decrease in leisure activity followed by a rise in sedentary behaviors and the degradation of eating habits have been observed. These changes have led to an increasing risk of developing metabolic diseases such as diabetes mellitus (DM) (1–3). In 2004, Wild et al. (4) estimated that 4.4% of the population worldwide will have diabetes in 2030 (representing 366 million people). Only 7 years later, Whiting et al. (5) assessed that 366 million people had diabetes and projected an increase to 552 million people by 2030. Various solutions exist to combat this increasing prevalence (6). Physical activity (PA) has been shown to be the main factor in primary prevention. Accordingly, studies show a decrease of 15–67% in the relative risk of developing DM (7–11). When associated with a healthy diet, PA is also considered to be an imperative component in the treatment of subjects with DM (12). Indeed, in the short term, appropriate exercise can decrease glycemia by burning the overflow of blood glucose to generate energy. Long term, PA can improve insulin sensitivity, glycemic control, systolic blood pressure, and weight loss. It can also increase ${\dot{V}}_{{\text{O}}_{2\text{ peak}}}$, reduce glycated hemoglobin and fasting plasma glucose, and finally, decrease the need for oral medications or insulin (13–18). All these improvements are considered sufficient to decrease the rate of diabetes complications (19) and the relative risk of all-cause mortality (20, 21). Nevertheless, due to the impaired glucose regulation, exercise duration and intensity must be considered attentively. Actually, long duration exercise without sugar intake may increase the risk of hypoglycemia. On the contrary, brief intense exercise may induce hyperglycemia requiring insulin intake. Underestimated energy expenditure may also lead to an underestimation of necessary medication. In these cases, exercise as a therapeutic tool must be precisely programed into daily life in order to see benefits in patients with DM.

The aim of this article is twofold. First, it presents the differences between the energy expenditure between of people with DM and healthy people. Second, it reports on the various methods for evaluating EE and their validity in subjects with DM.

## Energy Expenditure in Subjects with DM

In the literature, a simple model defines the daily total energy expenditure (TEE) as the sum of the basal energy expenditure (BEE), the thermic effect of food (TEF), and the activity energy expenditure (AEE). BEE is the major component of internal heat produced. BEE, which is defined as the energy expended to maintain minimal metabolic activities during a non-active period, is the main component of TEE (60–70% of TEE) (22). The TEF symbolizes the energy used by the body when it processes certain unrefined foods as lean meats, vegetables, and whole grains. AEE represents the energy expended through PA and volitional exercise and sports. AEE is the main parameter that allows modulation of TEE since it depends on the type of PA, its duration, and its intensity. Thus, in the sections below, we will present the differences between the three EE components in patients with DM and healthy people.

### BEE in Subjects with DM

Studies exploring BEE in patients with DM report no difference in absolute BEE (22–25). Nevertheless, this preliminary result could be explained by the sample heterogeneity of these studies. Indeed, after adjusting for age, sex, and fat free mass (FFM), it appeared that people with uncontrolled DM (HbA1c ≥ 8%) had a BEE 7.7% higher than healthy people (25–30).

Several possible physiological mechanisms may induce changes in BEE. FFM explains 65–90% of the interindividual variation of BEE in healthy adults (31). In subjects with DM, fasting blood glucose (FBG) may be another independent determinant of BEE (26–29). Indeed, studies found a 3–8% increase in BEE in patients with DM with high FBG (>10 mmol/l), which returns to the normal after insulin therapy (26, 29). These results are supported by the study of Ryan et al. (22) who reported no increase in the absolute and adjusted BEE in patients with a treated and stable DM. These results have been explained by two hypotheses. First, BEE may be increased by a rise in the loss of glucose in the urine (glycosuria) (29). It has been shown that hyperglycemia can increase glycosuria by 30–80 g/day, which corresponds to an energy loss of about 120–320 kcal/day (32). Finally, the second hypothesis indicated that people with impaired glucose regulation had an increase in fasting hepatic gluconeogenesis with the rise of glycemia (33). Gluconeogenesis is an important energy-consuming process that transforms free fatty acid into glucose causing by the decrease of insulin plasma level.

Overall, these studies suggest that the increase in glycosuria and/or gluconeogenesis caused by hyperglycemia seems to be behind the high BEE observed in patients with DM with poor glycemic control.

### Thermic Effect of Food

Studies examining the effects of DM on the TEF estimated a lower TEF in patients with insulin resistance than in healthy people (23, 34). Another study assessing insulin-induced thermogenesis (defined as the percentage of increase in metabolic rate during an insulin/glucose infusion) showed a decrease from subjects without DM (11.7%) to patients with impaired glucose tolerance (7.3%) and finally patients with DM (6.5%) (35). Thus, it appears that TEF decreases progressively with the development of DM. Indeed, TEF is negatively correlated with FBG and insulin concentrations (both are predictors of insulin resistance). The decrease in TEF may be induced by a reduced rate of glycogenesis in the skeletal muscle (36) and an impaired activation of the sympathetic nervous system (37). As discussed later in this report, TEF will not be studied further because it does not represent an adjustable component of TEE.

### Activity Energy Expenditure in Subjects with DM

A few studies directly compare the AEE in free-living conditions of patient with DM and a control group and indicate a lower AEE in patients with DM than in healthy people (694 vs 1,086 kcal/day, respectively) (24, 38). Taken together, the results of these studies suggest that people with DM present a total AEE less than that of healthy people. This result may be explained by either a lower amount of PA in subjects with DM and/or for a similar activity, by a difference in mechanical or metabolic efficiency.

Physical activity guidelines give the minimal amount of exercise required to maintain or to improve people’s health. These directives concern the general population as well as patients with DM. A first guideline recommends accumulating an AEE of at least 800 kcal/week or 600 MET.min/week to be sufficiently active (39). This recommendation conducted led to instructions for practicing PA at moderate intensity for a minimum of 30 min, 5 days/week or vigorous PA for a minimum of 20 min, 3 days/week, or accumulating 10,000 steps, daily or at least 5 days/week (40–42).

According to these guidelines, 31–54.6% of individuals with DM were considered inactive (no activity practiced) and 13–53.6% insufficiently active. In the general population, 5–62% over 45 years of age were defined as inactive and 9–43% insufficiently active. In addition, studies indicated a lower rate of patients with DM following the guidelines than in the general population (12.4–43% vs 55.5–87.2% in healthy people) (43–53). When the number of steps was observed, patients with DM without neuropathy walked 3,292–9,049 steps/day on average (54–59). Mitsui et al. (60) showed that 12–27.1% of healthy people aged over 45 years of age walk more than 10,000 steps/day while in Strycker et al. (56), no patients with DM followed this recommendation and only 15% walked 7,500–9,999 steps/day.

To further analyze the practice of PA, activities can be qualified by their type (leisure, recreational, domestic), by their intensity (light, moderate, vigorous), or by their duration. By questioning the activity preferences of 505 Mexican–American patients with DM, study reported that the most common activities were gardening (33.7%) and walking (31.8%) and the least frequent were jogging (2.4%), aerobic (2.3%), and swimming (1.3%) (44). Moreover, studies also reported that 33, 16, and 12% of patients with DM had at least one, two, and three or more regular activities in the month, respectively, and 71% achieved at least one 10-min period/day of moderate PA and 26% at least one 10-min period/day of vigorous PA (44, 61). By using a questionnaire, Kriska et al. (62) estimated a lower number of PA hours/week in men with DM than in healthy men (12.1 vs 22.9 h/week) and found no difference in women. By contrast, Ford and Herman (63) showed that patients with DM were equally likely to have engaged in PA. Based on indirect calorimetry (IC), studies noted a higher 24-h spontaneous PA (+10.5%) and total activity counts in healthy people and pre-patients with DM than in individuals with DM (25, 64). By contrast, Fontvieille et al. (23) concluded that there was no difference in spontaneous PA between the two groups.

The lower AEE observed in patients with DM may result from a smaller amount of PA accumulated throughout the day. However, other parameters such as metabolic and mechanical efficiency can also reduce energy expenditure. With equal level of activity, different AEE may reflect the different use of energy fuel. Indeed, the energetic equivalent of 1 l of oxygen changes according to the type of foodstuffs metabolized (in a simple model, 1 l of oxygen equals 5.05 and 4.48 kcal when carbohydrate and fat are oxidized, respectively). The respiratory quotient is an indicator of the fuel substrate used and it is equal to the ${\dot{V}}_{CO_{2}}$ divided by the ${\dot{V}}_{{\text{O}}_{2}}$ (respiratory quotient = 1 for carbohydrate oxidation only and 0.70 for fat oxidation only). During moderate-intensity exercise (40–50% of ${\dot{V}}_{{\text{O}}_{2\;\max}}$), studies found no significant difference between the respiratory quotient and ${\dot{V}}_{{\text{O}}_{2}}$ of patients with DM and healthy people (65, 66). In a second hypothesis, mechanical efficiency may be another parameter explaining the difference in AEE between patient with DM and healthy people. For instance, walking is one of the most convenient daily PA recommended for increasing TEE. Studies of patients with DM showed a decrease in comfortable walking speed, cadence, and stride length and an increase of plantar pressure at the heel, mid-foot, and first metatarsophalangeal joint as compared with the control group (67, 68). Moreover, it was also demonstrated that patients with DM have a less total concentric work in lower limb than healthy people (69), but more cocontraction in the muscles at the ankle and knee joint during the stance period (70). This altered gait pattern observed in patients with DM seems to reflect a stabilization strategy to compensate for the development of peripheral neuropathy (diminished sensory information and maximum strength of the lower limbs). At speeds of walking range from 0.6 to 1.6 m/s, Petrovic et al. (66) presented a general pattern of a higher cost of walking (express as milliliters per kilogram per minute) in diabetic patients with neuropathy (significant difference at all speed of walking) and in diabetic patients without neuropathy (significant difference at 1.4 and 1.6 m/s) in comparison with healthy subjects. However, Maiolo et al. (71) found no significant difference in net EE of walking (at three different intensities) between patients with DM and healthy people.

In light of the previous results, the lower AEE in subjects with DM seems to be due to a low level of daily PA with more low intensity activities and an energetic inefficiency during walking. Due to the complex nature of PA, AEE assessment requires precise and adapted tools. In the remainder of our review, we will describe potential tools (validated for use in daily life) that are suitable for research with patients with DM.

## Assessment of Energy Expenditure in Subjects with DM

### Methods for Evaluating BEE in Subjects with DM

Measure of BEE is typically taken by IC. Although these methods are very accurate, they require significant human and financial resources. Thus, equations have been proposed to simply estimate the BEE with variables such as age, height, or weight. Many of these equations were constructed with data from the general population and then tested in people with DM (Table 1).

**Table 1:** **Most popular equations to estimate basal energy expenditure (kcal/day) derived from the general population**.

Reference	Equation	Population base
Harris and Benedict (72)	*Female*: 655.1 + (9.6 × Wt) + (1.86 × ht) − (4.68 × *A*)	*n* = 239. Young, white, and lean
	*Male*: 66.5 + (13.75 × Wt) + (5.9 × ht) − (6.76 × *A*)	
Ganpule et al. (73)	[(48.1 × Wt) + (23.4 × ht) − (13.8 × *A*) + (547.3 × Sex) − 423.5]/4.186	*n* = 137. Healthy lean Japanese
Schofield (74)	*Female*: [(0.034 × Wt + 3.653) × 1,000]/4.186	*n* = unclear. Older obese subjects
	*Male*: [(0.048 × Wt + 3.538) × 1,000]/4.186	
Mifflin et al. (75)	*Female*: (10 × Wt) + (6.25 × ht) − (5 × *A*) − 161	*n* = 498. Lean and obese
	*Male*: (10 × Wt) + (6.25 × ht) − (5 × *A*) + 5	
Owen (76)	186 + (23.6 × FFM)	*n* = 104. Lean and obese, different ethnic
Bernstein et al. (77)	*Female*: 844 + (7.48 × Wt) − (0.42 × ht) − (3 × *A*)	*n* = 202. Obese
	*Male*: −1032 + (11 × Wt) + (10.2 × ht) − (5.8 × *A*)	

*Wt, weight (kg); ht, height (cm); *A*, age (years); Sex, 1 for male, 0 for female; BMI, body mass index (kg/m^2^); FFM, fat free mass (kg); FM, fat mass (kg)*.

The Harris–Benedict equation is most frequently used to estimate BEE in the general population, but studies in patients with DM have presented mixed results. Many studies show a significant overestimation of BEE with this equation (RMSE^1^ = 160–184 kcal/day) (29, 78–80). Huang et al. (30) also found an overestimation of 3.3% in men with DM, but an underestimation of −3.1% in women. Finally, Miyake et al. (81) and de Figueiredo Ferreira et al. (82) observed no significant difference with IC (MD^2^ = −19 to 42.3 kcal/day). With the Mifflin–St Jeor equation, studies in patients with DM show an under/overestimation of BEE by −126 to 160 kcal/day (80, 81, 83), or no significant difference (29, 78). The Ganpule equation presented no difference with the reference measure (bias^3^ = 4.5%) (79) or underestimated BEE by −110 kcal/day (81). Those developed by Schofield and Rodrigues showed no significant difference with the reference to subjects with DM [MD = 55 kcal/day (*p* = 0.065) and −17.6 kcal/day (*p* = 0.845), respectively]. The other equations tested in diabetic population overestimated the BEE (RMSE = 155–209 kcal/day, bias = 5.9–12.3%) except for those of Owen and Bernstein, which underestimated BEE (MD = −62 to −53.5 kcal/day for the Owen equation and bias = −11.6 to −13.9% for the Bernstein equation) (79–81).

To improve the BEE estimate, other equations taking into account the physiological specificities of subjects with DM were developed (Table 2) and compared with equations established for the general population, and with IC (Table 3). The Huang and Martin equations presented comparable coefficients of determination (*r*^2^ = 0.75 and 0.79, respectively) and had better results as compared with the Harris–Benedict equation (30, 80). Besides, de Figueiredo Ferreira et al. (83) showed an overestimation of BEE (MD = 115 kcal/day) in women with DM with this equation. The Ikeda and Martin equations presented a lower RMSE (103 and 136 kcal/day, respectively) than the other general equations (140–209 kcal/day) (79, 80). Finally, the Gougeon equation, taking into account the glycemic status, presented no significant difference with IC [MD = 7.4 kcal/day (*p* = 0.845) and bias = −0.5 and 1.6%] (29, 83).

**Table 2:** **Equations to estimate basal energy expenditure (kcal/day) derived from patient with diabetes or mixed population**.

Reference	Equation	Population base
Gougeon et al. (29)	375 + (85 × Wt) − (48 × FM) + (63 × FBG)	*n* = 65. Obese, with diabetes mellitus
Huang et al. (30)	71.767 − (2.337 × *A*) + (257.293 × Sex) + (9.996 × Wt) + (4.132 × ht) + (145.959 × DSI)	*n* = 1088. Obese, with and without DM
Martin et al. (80)	*Female*: 803.8 + (0.3505 × *A*) × (BMI − 34.524) − (135 × Race) + (15.866 × FFM) + (50.90 × DSI)	*n* = 166. Lean and obese, white and black, with and without DM
	*Male*: 909.4 + (0.3505 × *A*) × (BMI − 34.524) − (135 × Race) + (15.866 × FFM) − (9.10 × DSI)	
Ikeda et al. (79)	(10 × Wt) − (3 × *A*) + (125 × Sex) + 750	*n* = 68. Japanese, patients with type 1 and type 2 diabetes, lean and overweight

*Wt, weight (kg); ht, height (cm); *A*, age (years); Sex, 1 for male and 0 for female; BMI, body mass index (kg/m^2^); FFM, fat free Mass (kg); Race, 1 for black and 0 for white; DSI: 1 for patient with diabetes and 0 for healthy; FBG: fasting blood glucose (mM)*.

**Table 3:** **References and results of studies comparing predictive basal energy expenditure with the gold standard in patients with type 2 diabetes**.

Reference	Population	Equation	Results
Miyake et al. (81)	*n* = 30; healthy Japanese (*n* = 10), with IGT (*n* = 7) and with DM (*n* = 13)	Harris–Benedict	In DM—MD = −19 kcal/day
		Ganpule	In DM—MD = −110 kcal/day^‡^
		Schofield	In DM—MD = +55 kcal/day
		Owen	In DM—MD = −62 kcal/day
		Mifflin–St Jeor	In DM—MD = −126 kcal/day^‡^

Huang et al. (30)	*n* = 1,088; DM = 142; obese subjects with and without DM	Huang	*R*^2^ = 0.75
		Harris–Benedict	DM—bias = −3.1 to 3.3%^†^

Merghani et al. (78)	*n* = 80; obese subjects with DM (*n* = 40)	Mifflin–St Jeor	In DM—no significant difference (*p* = 0.164)
		Harris–Benedict	In DM—overestimate by 11%^‡^

de Figueiredo Ferreira et al. (83)	*n* = 28; sedentary Brazilian women	Harris–Benedict	MD = 42.3 kcal/day; bias = 5.9%
		Mifflin–St Jeor	MD = −69.6 kcal/day; bias = −2.6%
		Huang	MD = 115 kcal/day; bias = 11.3%^†^
		Owen	MD = −53.5 kcal/day; bias = −0.5%
		Gougeon	MD = 7.4 kcal/day; bias = 2.8%

Ikeda et al. (79)	*n* = 60; lean and overweight Japanese, patients with type 1 (*n* = 6), type 2 diabetes (*n* = 54)	Ikeda	RMSE = 103.0 kcal/day; bias = 4.8% (*ns*)
		Harris–Benedict	RMSE = 184.0 kcal/day; bias = 9.8% (*ns*)
		Ganpule	RMSE = 140.0 kcal/day; bias = 4.5% (*ns*)

Gougeon et al. (29)	*n* = 39; obese with DM (*n* = 32) and healthy obese subjects (*n* = 7)	Harris–Benedict	Bias = 11.6% in men^†^ and 4.8% in women^†^
		Owen	Bias = 2.3% in men and −5.3% in women^†^
		Mifflin–St Jeor	Bias = 1.8% in men and −0.7% in women
		Bernstein	Bias = −11.6% in men^†^ and −13.9% in women^†^
		Gougeon	Bias = −0.5% in men and 1.6% in women

Martin et al. (80)	*n* = 166; lean and obese subjects without DM (*n* = 97), with IGT (*n* = 22), with DM (*n* = 47)	Martin	RMSE = 136.0 kcal/day (*ns*)
		Harris–Benedict	RMSE = 160.1 kcal/day (*ns*)
		Mifflin–St Jeor	RMSE = 160.3 kcal/day (*ns*)
		Owen	RMSE = 163.2 kcal/day (*ns*)

*IGT, impaired glucose tolerance; DM, diabetes mellitus; bias, mean percentage error between estimated and measured BEE (%); RMSE, root mean squared error (kcal/day); MD, mean of difference between estimated and measured BEE (kcal/day); ^†^*p* < 0.05; ^‡^*p* < 0.001; *ns*, not specified*.

If BEE in subjects with DM cannot be accurately assessed using direct or IC, it can be adequately estimated using equations. The results of the aforementioned studies suggest that BEE in subjects with DM is better determined by using specific and adapted equations. Thus, the best results were obtained with the Gougeon equation and this may be due to the inclusion of the FBG as a variable.

### Evaluation of Free-Living Activity Energy Expenditure in Subjects with DM

Among the tools for assessing AEE, the doubly labeled water method and direct/IC are considered to be the gold standards. Although these different tools accurately estimated TEE and AEE, these methods cannot be used by a patient with DM to estimate his/her AEE and daily EE daily. For everyday use, many field evaluation tools for estimating AEE have been developed and tested in subjects with DM, such as diaries, questionnaires, or motion sensors. These methods and tools are often classified into two categories: subjective and objective methods.

#### Subjective Methods

Subjective methods include processes that usually require subjects to record their professional, home, and leisure activities. Among them, activity recall, logs, or questionnaires are declarative methods that provide a detailed account (nature, intensity, duration) of all daily PA. The AEE and TEE are then determined using the factorial method (84), where each activity intensity was weighted by its intensity expressed in Met, as it is referred to the compendium (85), then multiplied by its duration. As a reminder, one MET is equivalent to the BEE (1 MET = 3.5 ml/kg/min of oxygen consumption or 1 kcal/kg/h). At least, when added to the measured or predicted BEE, it is possible to predict the AEE and TEE with the sum of all activity estimates during the day. Thus, the use of questionnaires is widely reported in the literature on the topic because they are easy to use for large epidemiologic surveys. Nevertheless, few studies examined their validity with reference measures in subjects with DM.

The Modifiable Activity Questionnaire is a short survey created for the assessment of PA level (average hour/week of occupational and leisure activities over the past year) or energy expenditure in a variety of populations and age groups (86). Studies show that this questionnaire is both reliable and valid through direct comparisons with doubly labeled water (*r* = 0.75) (87) and accelerometer (*r* = 0.62) (86) in adults with DM. The International Physical Activity Questionnaire is most frequently used in epidemiologic research. It asks subjects about their PA during past week. Studies comparing the AEE estimated by this questionnaire with those estimated with accelerometer found a positive correlation in patients with DM with a correlation coefficient ranging from 0.24 (*n* = 143, *p* < 0.05) (88) to 0.53 (*n* = 31, *p* = 0.002) (89). The two questionnaires refer to the frequency and duration of activities and calculate an estimated AEE in kcal/week.

Subjective methods suffer from acknowledged limitations due to the subject’s ability to modify the information collected (90). Moreover, the use of the factorial method to assess the AEE may be inaccurate when applied to individuals with different fat mass or FMM. Indeed, the compendium was developed to identify different classes of PA and normalize MET intensities in healthy populations (85). Studies confirmed that the normally used 1 MET value overestimated the resting ${\dot{V}}_{{\text{O}}_{2}}$ (35%) and BEE (20%) in healthy adults and overestimated BEE in overweight and obese subjects (91, 92).

#### Objective Methods

Objective methods include tools that are based on physiological data (skin temperature or heart rate), mechanical data (pedometer, accelerometer, or inertial sensor), or a combination of both such as the SenseWear Armband (BodyMedia, Pittsburgh, PA, USA) or the Actiheart (Mini-Mitter Co., USA). The accurate measurement of TEE and AEE in subjects with DM is very challenging because, as demonstrated previously, patients with DM perform primarily low intensity PA, which may influence the assessment of AEE in several tools (93, 94). Therefore, these devices must be validated specifically for this population.

Few devices have been evaluated in people with DM. Mignault et al. (95) used the SenseWear Armband in six patients with DM for a 10-day free-living period to estimate their AEE and then compared it with the doubly labeled water based estimation. No significant difference was found between the two methods with a mean error of 78 kcal/day (*r* = 0.97). Machac et al. (94) evaluated the SenseWear Armband and the Omron HJ-720 (Omron Healthcare, Kyoto, Japan) under three walking conditions (3 km/h, 0%; 4 km/h, 0%; 5 km/h, 5%) in comparison with IC in 19 patients with DM. Their results showed a large overestimation with both devices at the lower speeds (MD = 70 and 81% at 3 km/h and 75 and 78% at 4 km/h for the Omron and Armband, respectively). On the contrary, both tools underestimated the AEE at 5 km/h, 5% (−7.3% for the Omron and −7.8% for the Armband).

As studies involving patients with DM are rare, the results of measurements for individuals with physical activities similar to those of patients with DM (low walking speed, light intensity exercise) could be enlightening. Studies in overweight and obese people without DM compared the Ormon and the SenseWear Armband with IC showing a similar overestimation of AEE during an exercise test (96, 97). In older adults, Mackey et al. (98) showed that the SenseWear Armband presented no difference from the gold standard for the assessment of TEE, but underestimated AEE by 18.5–26.8%. Colbert et al. (99) found RMSE of 185, 210, and 213 kcal/day for Actigraph (ActiGraph, LLC, USA), the SenseWear Armband, and NL-2000 (New-Lifestyles, Inc., Lee’s Summit, MO, USA), respectively. In another study, the Caltrac (Hemokinetics, Inc., Madison, WI, USA) underestimated TEE by −55 to −50% and no correlation was found with the double labeled water (100, 101). In their study of elderly men, Rafamantanantsoa et al. (102) compared TEE estimated by a heart rate monitor (Accurex Plus, Polar Electro Oy, Kempele, Finland) and an accelerometer (LifeCorder, Suzuken Co., Japan) with the doubly labeled water estimate. The heart rate method gives a better estimation of TEE (MD = 57 vs −542 kcal/day for accelerometry) but presented a higher intra-individual variation than the LifeCorder (coefficient of variation = 15 vs 7% for the accelerometer). As observed, objective methods suffer from acknowledged limitations. Accelerometry-based tools demonstrated poor accuracy at slow walking speeds and a decrease in precision with increasing body mass index (93). The device based on heart rate had a high variation in accuracy because the relation between heart rate and energy expenditure is not linear during rest and light intensity activity (103, 104). Yet, this range of intensities represented a major part of daily life activities in type 2 diabetic person.

Consequently, limited methods tested in people with DM may be used to self-check the calories burned daily. Currently, new technologies are accessible and marketed for estimating TEE or AEE and need to be tested in diabetic population.

### Future Method for Evaluating AEE in Subjects with DM

As previously discussed, different methods exist for the assessment of TEE or AEE, but few have been validated in people with DM. Future research should address the weaknesses of methods already tested or experiment with new devices, perhaps by combining several technologies.

Accelerometry showed higher error particularly during low intensity activities and cycling (99, 105, 106). In order to overcome this limitation, Bonomi et al. (107) demonstrated that the AEE estimate *via* accelerometry can be improved if it is combined with detection of the type of activity. These results are interesting because the use of data provided by only one accelerometer through a decision tree allows the correct detection of activity in 93% of cases (105). Kwapisz et al. (108) found similar result (91.7%) with a smartphone in detecting six activities of daily life. Several studies reported great accuracy in estimating AEE compared with IC (RMSE = 0.69–1.25 METs and MSE = 0.25 METs) and a lower error as compared to another method (MSE = 2.05 METs for the Actiheart) (109–111). Inertial sensors consisting of a three dimensions accelerometer and gyroscope and a magnetometer can be used to identify the type of activity performed like previous accelerometers (112). These methods based on activity recognition could be a reliable solution for estimating more precisely the AEE in subjects with DM. Finally, the Actiheart (combining an accelerometer and a heart rate monitor) demonstrated high accuracy in standardized and free-living conditions for the prediction of AEE in healthy adults, but need to be tested in people with DM (113, 114).

## Summary and Conclusion

This review emphasizes that there are differences between energy expenditure in patients with DM and healthy people. Patients with DM presented a higher BEE than healthy people. This difference seems to be due to an increase in FBG resulting in a higher glycosuria or gluconeogenesis. In addition, people with DM seem to have a lower AEE than healthy people. This review highlights that this lower AEE in patients with DM could be linked to a lower amount of activity (low compliance with PA recommendations) and the prevalence of low intensity activities. However, more studies should be conducted to determine the influence of diabetic altered gait on energy expenditure during PA. All these results demonstrate the need to develop adapted tools and methods to estimate free-living total energy expenditure in patients with DM. The results of this review indicate that there are valid equations for estimating BEE in patients with DM, but few of the methods tested give an accurate assessment of TEE and AEE in daily life. Other methods, such as those based on activity recognition with wearable sensors should be considered in the future to improve the estimation of daily TEE in subjects with DM. However, these possibilities need to be tested under everyday life conditions with patients with DM.

## Author Contributions

Drafting of manuscript: NC. Critical revision: NC, NP, TC, CV, and GD. Final approval of the version to be published: NC, NP, TC, CV, and GD. Agreement to be accountable for all aspects of the work: NC, NP, TC, CV, and GD.

## Conflict of Interest Statement

None of the authors of this manuscript have a direct financial relation with the commercial identities mentioned in this paper that might lead to a conflict of interests for any of the authors.

## Footnotes

^1^RMSE, root mean square error.

^2^MD, mean deviation.

^3^Bias, mean percentage error between estimated and measured BEE.

## Abbreviations

AEE, activity energy expenditure; BEE, basal energy expenditure; DM, diabetes mellitus; IC, indirect calorimetry; PA, physical activity; ${\dot{V}}_{CO_{2}}$, rate of carbon dioxide production; ${\dot{V}}_{O_{2}}$, rate of oxygen consumption; TEE, total energy expenditure; TEF, thermic effect of food.
